# GABAergic neurons of anterior thalamic reticular nucleus regulate states of consciousness in propofol‐ and isoflurane‐mediated general anesthesia

**DOI:** 10.1111/cns.14782

**Published:** 2024-06-03

**Authors:** Rulan Yi, Shiyu Cheng, Fuwang Zhong, Dan Luo, Ying You, Tian Yu, Haiying Wang, Liang Zhou, Yu Zhang

**Affiliations:** ^1^ Department of Anesthesiology Affiliated Hospital of Zunyi Medical University Zunyi China; ^2^ Key Laboratory of Anesthesia and Organ Protection (Zunyi Medical University), Ministry of Education Zunyi Medical University Zunyi China; ^3^ Key Laboratory of Brain Science Zunyi Medical University Zunyi China; ^4^ Guizhou Key Laboratory of Anesthesia and Organ Protection Zunyi Medical University Zunyi China

**Keywords:** anterior thalamic reticular nucleus, general anesthesia, loss of consciousness (LOC), parvalbumin, somatostatin

## Abstract

**Background:**

The thalamus system plays critical roles in the regulation of reversible unconsciousness induced by general anesthetics, especially the arousal stage of general anesthesia (GA). But the function of thalamus in GA‐induced loss of consciousness (LOC) is little known. The thalamic reticular nucleus (TRN) is the only GABAergic neurons‐composed nucleus in the thalamus, which is composed of parvalbumin (PV) and somatostatin (SST)‐expressing GABAergic neurons. The anterior sector of TRN (aTRN) is indicated to participate in the induction of anesthesia, but the roles remain unclear. This study aimed to reveal the role of the aTRN in propofol and isoflurane anesthesia.

**Methods:**

We first set up c‐Fos straining to monitor the activity variation of aTRN^PV^ and aTRN^SST^ neurons during propofol and isoflurane anesthesia. Subsequently, optogenetic tools were utilized to activate aTRN^PV^ and aTRN^SST^ neurons to elucidate the roles of aTRN^PV^ and aTRN^SST^ neurons in propofol and isoflurane anesthesia. Electroencephalogram (EEG) recordings and behavioral tests were recorded and analyzed. Lastly, chemogenetic activation of the aTRN^PV^ neurons was applied to confirm the function of the aTRN neurons in propofol and isoflurane anesthesia.

**Results:**

c‐Fos straining showed that both aTRN^PV^ and aTRN^SST^ neurons are activated during the LOC period of propofol and isoflurane anesthesia. Optogenetic activation of aTRN^PV^ and aTRN^SST^ neurons promoted isoflurane induction and delayed the recovery of consciousness (ROC) after propofol and isoflurane anesthesia, meanwhile chemogenetic activation of the aTRN^PV^ neurons displayed the similar effects. Moreover, optogenetic and chemogenetic activation of the aTRN neurons resulted in the accumulated burst suppression ratio (BSR) during propofol and isoflurane GA, although they represented different effects on the power distribution of EEG frequency.

**Conclusion:**

Our findings reveal that the aTRN GABAergic neurons play a critical role in promoting the induction of propofol‐ and isoflurane‐mediated GA.

## Graphical abstract

Optogenetic and chemogenetic activation of the GABAergic neurons in the anterior sector of the thalamic reticular nucleus (aTRN) promote the induction of propofol‐ and isoflurane‐mediated general anesthesia.

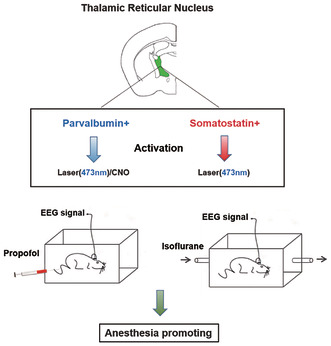

## 1 INTRODUCTION

General anesthesia (GA) mediated by different kinds of general anesthetics, such as propofol, isoflurane and sevoflurane, causes reversible loss of consciousness (LOC).^1^, ^2^, ^3^ However, the neural mechanism of GA‐induced loss and recovery of consciousness remains unclear. Previous studies suggested the critical roles of the thalamus system in regulation of GA.^1^, ^4^, ^5^ The functional connectivity of thalamocortical networks was considered as an essential regulator of consciousness loss mediated by GA.^4^, ^5^, ^6^, ^7^, ^8^ Numerous studies showed that thalamocortical functional connectivity was disrupted during GA‐induced unconsciousness in animals and humans.^9^, ^10^, ^11^, ^12^, ^13^ Moreover, multiple nuclei in the thalamus, such as the paraventricular thalamus (PVT), the central medial thalamic nucleus (CMT) and their related neural circuits, have been reported to play important roles in the regulation of states of consciousness during GA, especially the recovery period after GA.^14^, ^15^, ^16^, ^17^, ^18^, ^19^, ^20^, ^21^ A recent study also revealed that the ventral posteromedial nucleus (VPM) of the thalamus actively promoted arousal of GA, which is independent of the process of anesthesia.^22^ These findings suggested the importance of the thalamus in the recovery of consciousness after GA. However, the roles and mechanisms of the thalamus in GA‐induced consciousness loss were barely known.

The thalamic reticular nucleus (TRN) is the only GABAergic neurons‐composed nucleus in the thalamus, which is the main source of inhibition for the thalamus.^23^, ^24^ The TRN is considered as the “guardian of the gateway” in the thalamocortical circuit, which modulates thalamocortical oscillations to regulate the related brain functions, including sensation, attention, sleep, arousal and cognition.^25^, ^26^, ^27^, ^28^, ^29^, ^30^ The TRN was reported to synchronize brain‐wide network activity.^25^, ^26^, ^31^ Brief optogenetic stimulation of TRN neurons induces spindles in the cortex,^32^ whereas the alpha oscillations (8–12 Hz) in the cortex is identified as a characteristic of propofol‐induced anesthesia,^33^, ^34^, ^35^, ^36^, ^37^ which indicated the involvement of TRN in GA regulation. Furthermore, manipulation of the basal forebrain (BF)‐TRN or the locus coeruleus (LC)‐TRN pathway affected the progress of isoflurane‐ or propofol‐mediated GA.^38^, ^39^ The TRN is anatomically divided into several sectors distributed along the plane of the nucleus, and each sector forms distinct neural circuits which are composed of the specific cortical afferents and the thalamic target of TRN neurons.^40^ The anterior sector of the TRN (aTRN) receives the projections from the prefrontal cortex (PFC)^41^ and sends mainly outputs to the thalamic nonspecific nuclei, such as the thalamic mediodorsal nucleus (MD) and the VPM^40^ to play a part the regulation of the corticothalamic and thalamocortical activities.^41^ These findings suggested that the aTRN neurons may participate in GA‐induced loss and recovery of consciousness, but the roles remain unclear.

In the present study, we investigated how GABAergic neurons in aTRN regulate GA induced by propofol and isoflurane. c‐Fos staining was set up to monitor the activity variation of aTRN neurons during distinct stages of GA. Subsequently, optogenetic or chemogenetic activation of aTRN GABAergic neurons was performed to clarify the function of aTRN during propofol‐/isoflurane‐mediated GA. Taken together, our findings show a critical role of aTRN GABAergic neurons in the modulation of propofol‐ and isoflurane‐induced unconsciousness.

## 2 MATERIALS AND METHODS

### 2.1 Animals

Adult (9–12 weeks, 22–25 g) male mice were used for all experiments. C57BL/6J mice were purchased from Changsha Tianqin Technology Co., Ltd. (Changsha, China). SST‐IRES‐Cre (Strain #013044) and PV‐IRES‐Cre (Strain #017320) mice were from the Jackson Laboratory (USA). VGAT‐tdTomato mice were generated in our laboratory by mating VGAT‐IRES‐Cre mice (the Jackson Laboratory, Strain #028862) with Ai9 mice (the Jackson Laboratory, Strain #007909). Mice were maintained at the Experimental Animal Center of Zunyi Medical University (License Key, SYXK (Qian) 2021‐0004) and kept at room temperature (23 ± 2°C) on a 12:12 h light/dark cycle (light on at 7:00 p.m.). Age‐ and weight‐matched mice were randomly into the control or experimental groups respectively. All behavior and EEG tests were developed between 7:00 a.m. and 19:00 p.m.

All animal procedures were complied with the ARRIVE guidelines and were designed and conducted in accordance with the National Institutes of Health guide for the care and use of Laboratory animals (NIH Publications No. 8023, revised 1978) and the guidelines set forth by Zunyi medical university and the Guide for the Care and Use of Laboratory Animals of China (No. 14924, 2001).

### 2.2 Antibodies and reagents

Isoflurane (R510‐22) was provided by RWD Life Sciences (Shenzhen, China). Propofol (H20060314) was from AstraZeneca S.p.A. (Italy). Ibotenic acid (IBO, HY‐N2311) and Clozapine N‐oxide (CNO, HY‐17366) were purchased from MedChemExpress LLC (United States). Antibody against c‐Fos (226008, RRID:AB_2891278) was purchased from Synaptic Systems (Germany). The Somatostatin antibody (SAB4502861, RRID:AB_10747468) was obtained from Sigma‐Aldrich (United States). The Parvalbumin antibodies (PV27, RRID:AB_2631173 and 235, RRID:AB_10000343) were purchased from Swant (Switzerland). Alexa Fluor‐conjugated secondary antibodies were purchased from Thermo Fisher Scientific (A‐11073, RRID:AB_2534117; A‐11008, RRID:AB_143165; A‐11012, RRID:AB_2534079 and A‐11005, RRID:AB_141372).

### 2.3 Stereotaxic surgery

Mice were placed on a stereotaxic apparatus (RWD Life Science, Shenzhen, China) and anesthetized with 1.4% isoflurane. For local analgesia, lidocaine was subcutaneously injected before the skull surface exposure.

For optogenetic activation experiments, 150 nL rAAV‐EF1α‐DIO‐hChR2(H134R)‐mCherry (ChR2) or rAAV‐EF1α‐DIO‐mCherry (control) virus (Brain‐VTA, Wuhan, China) was unilaterally injected into the aTRN ([AP]: −0.53 mm, [ML]: +1.35 mm, [DV]: −3.65 mm, 20 nL/min) of PV‐IRES‐Cre or SST‐IRES‐Cre mice through a glass micropipette (1‐mm glass stock, tapering to a 10–20‐μm tip) using amicro‐syringe pump., followed by an optical fiber (Inper Ltd, Hangzhou, China) plantation into the same side of aTRN. After virus injection, the micropipette was indwelled in place for 10 min before slow retrieval. After virus injection, the EEG electrodes were placed on the skull (AP: +1.0 mm, ML: ±1.5 mm; AP: −3.5 mm, ML: ±1 mm) and firmed with skull screws and dental cement. All tests were performed after 3 weeks.

For chemogenetic activation tests, 150 nL rAAV‐hSyn‐DIO‐hM3D(Gq)‐EGFP (hM3Dq), or rAAV‐hSyn‐DIO‐EGFP (control) virus (Brain‐VTA, Wuhan, China) was unilaterally injected into the aTRN ([AP]: −0.53 mm, [ML]: +1.35 mm, [DV]: −3.65 mm, 20 nL/min) of PV‐IRES‐Cre or SST‐IRES‐Cre mice, followed by the placement of EEG electrodes on the skull (AP: +1.0 mm, ML: ±1.5 mm; AP: −3.5 mm, ML: ±1 mm).^40^

For lesion tests, equal volume of IBO (200 nL/side) and saline (as control) were bilaterally injected into aTRN region ([AP]: −0.53 mm, [ML]: ±1.35 mm, [DV]: −3.65 mm) of VGAT‐tdTomato mice. The pipette was hold at the injection site for 10 min to allow IBO to diffuse and was then slowly withdrawn. LORR and RORR measurements were performed after 7 days.

### 2.4 Behavioral tests

The induction time of loss of righting reflex (LORR) and the duration time recovery of righting reflex (RORR) are used as standard indices of GA induction and emergence in mice, respectively.

For propofol anesthesia, a single dose of 20 mg/kg propofol was administered through the caudal vein. The duration of anesthesia was defined as the RORR time. For isoflurane anesthesia, mice were placed in a recording chamber (RWD Company, Shenzhen, China) and incubated with 1.4% isoflurane in 100% oxygen at a rate of 1 L/min. An anesthesia monitor (Vamos; Drager Company, Germany) was connected to detect the concentration of isoflurane in the anesthesia chamber and an electric blanket with a rectal temperature probe was used to the bottom of the anesthesia chamber and was controlled at 37.5°C in the whole experiment. The interval between the starting point of isoflurane application and the time point at which mice lost the righting reflex was regarded as the latency to LORR. The mice were kept anesthetized for 20 min. After that, we turn off isoflurane infusion and immediately exhaust the remaining isoflurane in the chamber. The duration from the end of isoflurane infusion to RORR of mice was defined as RORR time. There was at least 7 days rest between light‐on and light‐off or CNO and saline treatment and isoflurane or propofol administration in the same mouse.

For optogenetic experiments, blue laser (473 nm, 5 mw, 10 Hz, 10 ms) stimulation was delivered to the mice during induction and emergence of GA. For induction, opto‐stimulation was administered with isoflurane inhalation and continued until the mice achieved LORR. For emergence, the mice were optically stimulated from the cessation of isoflurane/propofol application until RORR. In the chemogenetic groups, Clozapine N‐oxide (CNO) (1 mg/mL, 1 mg/kg, i.p.) or saline (0.9%, equal volume, i.p.) was injected 1 h before the behavioral tests and EEG recording. The LORR, RORR, and EEG were recorded under isoflurane and propofol application. In the lesion experiments, we recorded the LORR under isoflurane anesthesia and RORR under propofol/isoflurane anesthesia. Immunofluorescence analysis was performed on all mice to verify the position and efficiency of viral transfection after all tests were completed.

### 2.5 EEG recording

EEG was recorded using a multichannel signal acquisition system (Appolo, Bio‐Signal Technologies, USA) and then digitized and analyzed using the Spike2 software (Cambridge Electronic Design, Cambridge, UK). EEG signals were recorded for 5 min before induction and were continuously recorded until recovery from propofol or isoflurane anesthesia. Relative powers in the different frequency bands were computed by averaging the signal power across the frequency range of each band (delta (δ): 1–4 Hz, theta (θ): 4–8 Hz, alpha (α): 8–12 Hz, beta (β): 12–25 Hz, and gamma (γ): 25–60 Hz) and then dividing by the total power from 1 to 60 Hz. MATLAB (R2020a; MathWorks, Natick, MA, USA) and customized MATLAB codes were used for burst suppression ratio (BSR) calculation.^42^ We calculated the BSR in the LORR and RORR period of isoflurane anesthesia and the RORR period of propofol anesthesia in the optogenetic experiments. In the chemogenetic experiments, we calculated the BSR from the administration of isoflurane/propofol to the RORR of the mice.

### 2.6 In vitro electrophysiological recording

Three weeks after virus transfection, the brains of PV‐IRES‐cre or SST‐IRES‐cre mice were dissected out and incubated in oxygenated (95% O_2_/5% CO_2_) ice‐cold slicing buffer containing 234 sucrose, 3 KCl, 1.25 NaH_2_PO_4_, 26 NaHCO_3_, 0.5 CaCl_2_, 10 MgSO_4_, and 10 glucose (mmol/L, respectively). Coronal slices containing aTRN were cut as 240 μm thick with a vibratome (Thermo HM650V, USA) and incubated at 32°C for 20 min in artificial cerebrospinal fluid (ACSF) containing 126 NaCl, 3 KCl, 26 NaHCO_3_, 1.25 NaH_2_PO_4_, 1 MgSO_4_, 1.2 CaCl_2_, and 10 glucose (mmol/L, respectively, pH 7.4), aerated with 95% O_2_/5% CO_2_. Then, the slices were transferred to the recording chamber and perfused with oxygenated ACSF (1.5–2 mL/min) for 60 min at room temperature.

Whole‐cell recording was performed using micropipettes (3–5 _M_Ω) prepared by a horizontal puller (P‐97, Sutter Instruments). The pipette solution consisted of 135 K‐MeSO_4_, 5 KCl, 0.5 CaCl_2_, 5 HEPES 0.3 EGTA, 2 ATP‐Mg, and 0.5 GTP‐Na, (mmol/L, respectively, pH 7.4). A current clamp was used to assess the electrophysiological features in response to TRN^PV^ or TRN^SST^ neuron activation.

### 2.7 Immunohistochemistry

Mouse brains were collected after behavioral tests and EEG recordings. The brain tissues were fixed in 4% PFA and *dehydrated* in 30% sucrose, then frozen in OCT and sectioned at 20 μm using a freezing microtome (Leica CM1900, Germany). Sections containing aTRN were blocked in blocking buffer (10% goat serum, 1% BSA, and 0.3% Triton in PBS) for 2 h at room temperature (RT), followed by incubating with primary antibodies overnight at 4°C and secondary antibodies for 2 h at RT. The primary antibodies used were 1:1000 for c‐Fos, 1:200 for SST, and 1:1000 for PV. All secondary antibodies were diluted to 1:500. Sections were mounted using the DAPI Fluoromount‐G (Southern Biotech). All images were captured by a virtual microscopy system (Olympus BX63) and a laser confocal fluorescence microscope (Olympus FV1000). Only data from mice in which the AAV infection and the location of the optical fiber were confirmed were included. In this study, mice with poor AAV expression, misplaced optical fibers, or loose electrodes were excluded from further analysis.

### 2.8 Statistical analysis

We utilized GraphPadPrism 8.0 (GraphPad Software Inc., USA) for statistical analyses. All data were subject to tests for normality. The cell counting of c‐Fos staining among the wake, propofol/isoflurane and recovery groups was analyzed by one‐way ANOVA. IBO experiments were detected using the independent‐samples *t*‐tests. Independent‐samples *t*‐test was also applied in the analysis of LORR and RORR times between the control and IBO groups. Furthermore, paired Student's *t*‐tests were used to analyze differences in the change in LORR and RORR times, the changes of EEG power bands and BSR for chemogenetic and optogenetic experiments within group (CNO vs. saline or light on vs. light off). Moreover, one‐way ANOVA was used to the comparison of LORR times and RORR times and the changes of EEG power bands in optogenetic or chemogenetic experiments between groups (mCherry‐light on vs. ChR2‐light on or EGFP‐CNO vs. M3‐CNO). All data are shown as mean ± SD. *p* < 0.05 was considered statistically significant. “*n*” refers the number of animals we tested in the corresponding experiments.

## 3 RESULTS

### 3.1 aTRN GABAergic neurons are activated during the induction of GA

**FIGURE 1:**
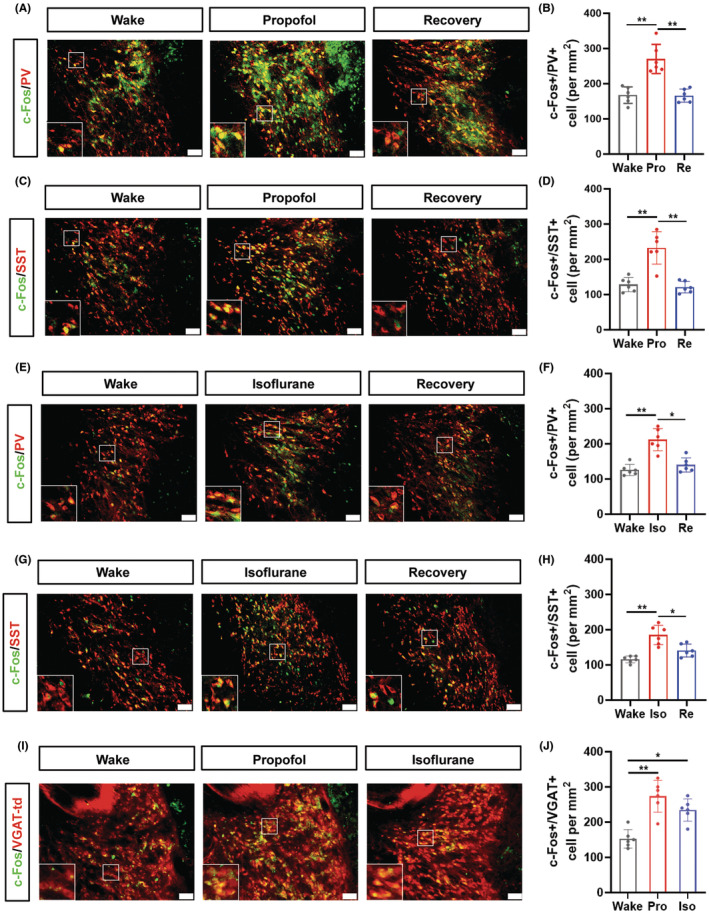
c‐Fos expression in aTRN during propofol and isoflurane anesthesia. (A) Representative images of c‐Fos/PV double‐staining in wake, propofol and recovery groups in aTRN (scale bar, 50 μm). (B) Quantification of c‐Fos^+^/PV^+^ number (per mm^2^) in aTRN (wake: 167 ± 23; propofol: 270 ± 41; recovery: 166 ± 18; *n* = 6). (C) Representative images of c‐Fos/SST double‐staining in wake, propofol and recovery groups in aTRN (scale bar, 50 μm). (D) Quantification of c‐Fos^+^/SST^+^ number (per mm^2^) in aTRN (wake: 128 ± 20; propofol: 232 ± 46; recovery: 121 ± 16; *n* = 6). (E) Representative images of c‐Fos/PV double‐staining in wake, isoflurane and recovery groups in aTRN (scale bar, 50 μm). (F) Quantification of c‐Fos^+^/PV^+^ number (per mm^2^) in aTRN (wake: 126 ± 16; isoflurane: 212 ± 31; recovery: 140 ± 20; *n* = 6). (G) Representative images of c‐Fos/SST double‐staining in wake, isoflurane and recovery groups in aTRN (scale bar, 50 μm). (H) Quantification of c‐Fos^+^/SST^+^ number (per mm^2^) in aTRN (wake: 116 ± 10; isoflurane: 185 ± 28; recovery: 141 ± 18; *n* = 6). (I) Representative images of c‐Fos expression in wake, propofol and isoflurane groups in aTRN of VGAT‐td mice (scale bar, 50 μm). (J) Quantification of c‐Fos^+^/VGAT^+^ number (per mm^2^) in aTRN (wake: 151 ± 26; propofol: 273 ± 45; recovery: 224 ± 25; *n* = 6). One‐way ANOVA: (B, D, F, H, J). *p < 0.05, **p < 0.01.

It is reported that TRN is composed of GABAergic neurons, which nearly all express PV and partly express SST.^43^ PV/SST double staining was set up to confirm the composition of aTRN. The co‐expression proportion of PV and SST neurons in TRN is about 40.5%, and the proportion of independent expression of PV or SST neurons is about 38% and 21.5%, respectively (Figure S1A,B). Furthermore, VGAT‐tdTomato (VGAT‐td) mice were used to monitor the proportion of PV or SST neurons in aTRN and we found that the proportion of PV is about 80.6%, whereas the proportion of SST is 56.8% (Figure S1C,D). Our data showed that about 40% of aTRN neurons are PV^+^/SST^+^ double positive, which is different from the separated PV/SST expression in cortical GABAergic neurons (Figure S1E). These findings indicated the possible distinct functions of PV and SST neurons in aTRN.

c‐Fos expression was used as an indicator to monitor the activity of aTRN neurons during the state of wakefulness, anesthesia, and recovery from anesthesia. Compared to wakefulness, increased number of PV^+^/c‐Fos^+^ cells was observed during propofol anesthesia, whereas PV^+^/c‐Fos^+^ cell number was decreased after recovery of consciousness (Figure 1A,B). Concurrently, similar accumulated SST^+^/c‐Fos^+^ expression was found in the propofol anesthesia groups compared to the wakefulness and recovery stages (Figure 1C,D). These data indicate that both PV and SST neurons in aTRN are activated during propofol anesthesia.

We next checked the activity of aTRN neurons during isoflurane anesthesia. Consistent with the propofol treatment, the number of both PV^+^/c‐Fos^+^ and SST^+^/c‐Fos^+^ cells in aTRN was increased during isoflurane anesthesia and decreased in the recovery period (Figure 1E–H). At last, we used VGAT‐td mice to confirm that the td‐tomato‐labeled TRN neurons are activated during both propofol and isoflurane anesthesia (Figure 1I,J). These findings suggest that both PV and SST‐expressed GABAergic neurons in aTRN may involve in the regulation of distinct stages of GA. Next, optogenetic and chemogenetic tools are used to specifically manipulate the activity of PV or SST‐expressed aTRN GABAergic neurons (aTRN^PV^ or aTRN^SST^) to clarify its precise function in GA.

### 3.2 Optogenetic activation of aTRN^PV^ Neurons delays the emergence of propofol anesthesia

**FIGURE 2:**
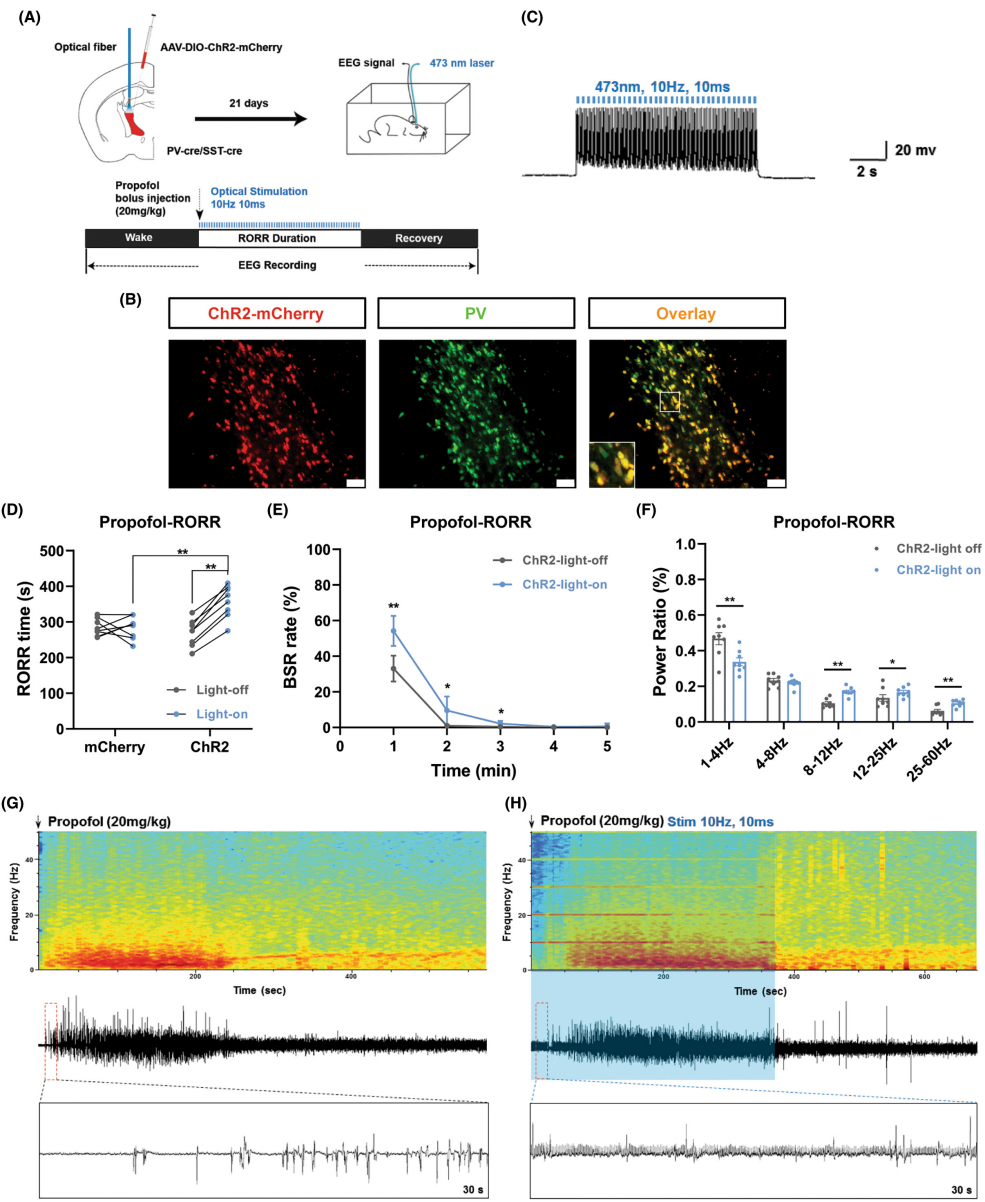
Optogenetic activation of aTRN^PV^ Neurons delays the emergence of propofol anesthesia. (A) Up: schematic diagram of virus injection into aTRN and optic fiber and EEG electrode implantation. Down: schematic diagram of RORR and EEG recording during propofol anesthesia by single dose of 20 mg/kg injection. (B) Representative images of optogenetic virus (ChR2‐mCherry, red) expression in TRN^PV^ neurons (green, scale bar, 50 μm). (C) Ex vivo electrophysiology of ChR2 virus action in aTRN^PV^ neurons. (D) The effect of aTRN^PV^ neuron activation by blue laser on RORR time under propofol anesthesia (mCherry‐light‐off: 284.75 ± 23.89 s; mCherry‐light‐on: 283.63 ± 31.66 s; ChR2‐light‐off: 269.50 ± 37.45 s; ChR2‐light‐on: 357.38 ± 45.36 s; *n* = 8). (E) The effect of aTRN^PV^ neuron activation by blue laser on BSR during the RORR period of propofol anesthesia (*n* = 8). (F) The power distribution of EEG frequency bands in ChR2‐light off or ChR2‐light on group under propofol anesthesia (*n* = 8). (G, H) Representative EEG wave forms and spectrograms EEG power of ChR2‐light off or ChR2‐light on group under propofol anesthesia. Paired Student's *t*‐tests and One‐way ANOVA: (D). Paired Student's *t*‐tests: (E, F). *p < 0.05, **p < 0.01.

First, we used optogenetic tools to access the roles of aTRN^PV^ neurons in propofol anesthesia. rAAV‐EF1α‐DIO‐hChR2‐mCherry virus (ChR2) was unilaterally injected into aTRN of the PV‐IRES‐Cre mice, followed by an optical fiber plantation to stimulate aTRN^PV^ neurons (Figure 2A,B and Figure S2A). During the induction and emergence periods of anesthesia, continuous blue laser stimulation was applied to activate the aTRN^PV^ neurons. The stimulation efficiency was monitored by electrophysiological recording in the ex vivo brain slices (Figure 2C).

A single dose (20 mg/kg) of propofol was used to reveal the effect of aTRN^PV^ neuron activation on propofol‐induced anesthesia. The tail vein injection of 20 mg/kg propofol caused an immediate coma in mice; therefore, we can evaluate the effect of aTRN^PV^ neurons on the emergence of propofol anesthesia (RORR). Optogenetic activation of aTRN^PV^ neurons prolonged the RORR time of propofol anesthesia (Figure 2D). EEG recordings were set up to further assess how aTRN^PV^ neurons affect propofol anesthesia. In the ChR2‐expressing mice, the burst suppression ratio (BSR) was notably increased during propofol anesthesia after aTRN^PV^ neuron activation (Figure 2E), which indicated the deeper anesthesia.^42^ Furthermore, optogenetic activation of aTRN^PV^ neurons caused a lower percentage of δ wave (1–4 Hz) and an higher percentage of α wave (8–12 Hz), β wave (12–25 Hz) and γ wave (25–60 Hz) during the emergence period of propofol anesthesia (Figure 2F–H and Figure S2B,C).

### 3.3 Optogenetic activation of aTRN^PV^ Neurons promotes the induction and delays the emergence of isoflurane anesthesia

**FIGURE 3:**
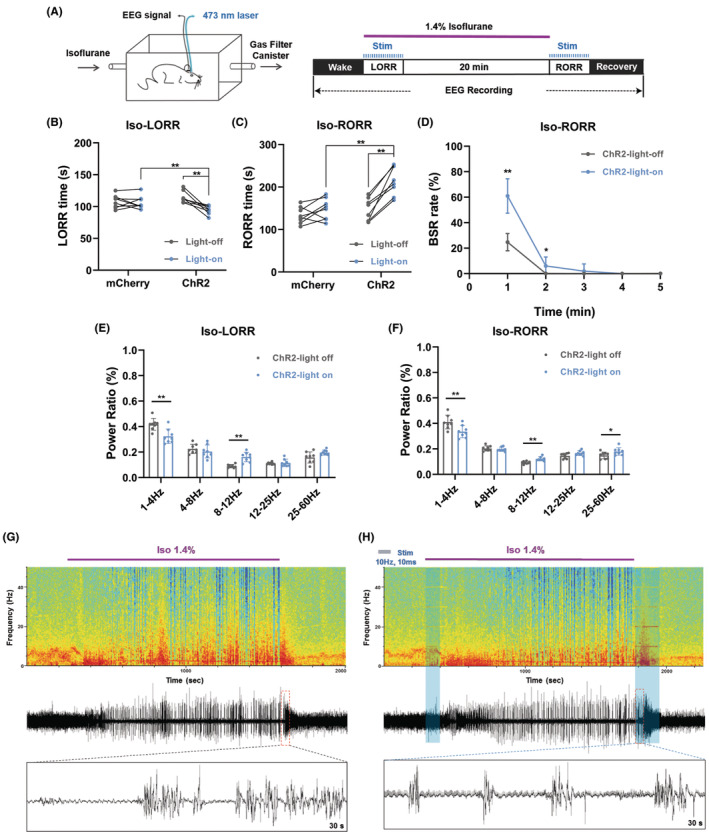
Optogenetic activation of aTRN^PV^ Neurons promotes the induction and delays the emergence of isoflurane anesthesia. (A) Schematic diagram of LORR, RORR and EEG recording during 1.4% isoflurane anesthesia. (B, C) The effect of aTRN^PV^ neuron activation by blue laser on LORR and RORR time under 1.4% isoflurane anesthesia (LORR: mCherry‐light‐off: 107.63 ± 10.27 s; mCherry‐light‐on: 106.12 ± 10.19 s; ChR2‐light‐off: 114.88 ± 8.90 s; ChR2‐light‐on: 94.50 ± 6.61 s; RORR: mCherry‐light‐off: 133.25 ± 19.40 s; mCherry‐light‐on: 148.00 ± 25.21 s; ChR2‐light‐off: 146.25 ± 26.67 s; ChR2‐light‐on: 214.87 ± 33.45 s; *n* = 8). (D) The effect of TRN^PV^ neuron activation by blue laser on BSR during the RORR period of 1.4% isoflurane anesthesia (*n* = 8). (E, F) The power distribution of EEG frequency bands in ChR2‐light off or ChR2‐light on group under 1.4% isoflurane anesthesia (*n* = 8). (G, H) Representative EEG wave forms and spectrograms EEG power of ChR2‐light off or ChR2‐light on group under 1.4% isoflurane anesthesia. Paired Student's *t*‐tests and One‐way ANOVA: (B, C). Paired Student's *t*‐tests: (D–F). *p < 0.05, **p < 0.01.

We further tested the effect of aTRN^PV^ neuron activation on isoflurane anesthesia (Figure 3A). Activation of aTRN^PV^ neurons by blue laser stimulation shortened the induction time (LORR) and prolonged the emergence time (RORR) of isoflurane anesthesia (Figure 3B,C). In the ChR2‐expressing mice, activation of aTRN^PV^ neurons increased the BSR in the emergence period of isoflurane anesthesia (Figure 3D), which is consistent with the propofol anesthesia (Figure 2E). EEG recordings also showed decreased δ wave (1–4 Hz) proportion and increased α wave (8–12 Hz) proportion during the induction of isoflurane anesthesia in the ChR2‐expressing mice after blue laser stimulation (Figure 3E,G,H and Figure S2D,E). Meanwhile, decreased δ wave (1–4 Hz) proportion and increased proportion of α wave (8–12 Hz) and γ wave (25–60 Hz) were observed during the emergence period while aTRN^PV^ neurons were activated by blue laser (Figure 3F–H, Figure S2F,G). Taken together, our data indicate that optogenetic activation of aTRN^PV^ neurons promotes the induction of isoflurane anesthesia and delays the recovery of both propofol and isoflurane anesthesia.

### 3.4 Optogenetic activation of aTRN^SST^ Neurons decelerates the emergence of propofol anesthesia

**FIGURE 4:**
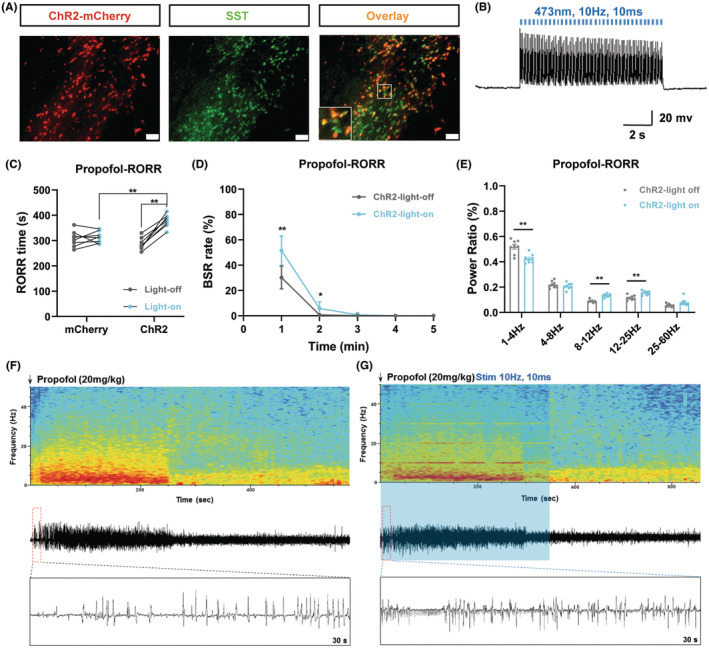
Optogenetic activation of aTRN^SST^ Neurons decelerates the emergence of propofol anesthesia. (A) Representative images of optogenetic virus (ChR2‐mCherry, red) expression in aTRN^SST^ neurons (green, scale bar, 50 μm). (B) Ex vivo electrophysiology of ChR2 virus action in aTRN^SST^ neurons. (C) The effect of aTRN^SST^ neuron activation by blue laser on RORR time under propofol anesthesia (mCherry‐light‐off: 307.00 ± 30.87 s; mCherry‐light‐on: 313.25 ± 22.38 s; ChR2‐light‐off: 286.88 ± 23.91 s; ChR2‐light‐on: 378.25 ± 24.62 s; *n* = 8). (D) The effect of aTRN^SST^ neuron activation by blue laser on BSR during the RORR period of propofol anesthesia (*n* = 8). (E) The power distribution of EEG frequency bands in ChR2‐light off or ChR2‐light on group under propofol anesthesia (*n* = 8). (F, G) Representative EEG wave forms and spectrograms EEG power of ChR2‐light off or ChR2‐light on group under propofol anesthesia. Paired Student's *t*‐tests and One‐way ANOVA: (C). Paired Student's *t*‐tests: (D, E). *p < 0.05, **p < 0.01.

Next, we generated ChR2‐transfected mice to access the roles of aTRN^SST^ neurons in propofol anesthesia. rAAV‐EF1α‐DIO‐hChR2‐mCherry virus (ChR2) was unilaterally injected into aTRN of the SST‐IRES‐cre mice, followed placement of an optical fiber in the same position (Figures 2A and 4A, Figure S3A). Electrophysiological recording in the ex vivo brain slices confirmed the activation of aTRN^SST^ neurons (Figure 4B).

After 20 mg/kg propofol injection into the mice, the effect of optogenetic activation of aTRN^SST^ neurons on RORR, and the EEG signature of propofol anesthesia was detected. We found that optogenetic activation of aTRN^SST^ neurons prolonged the RORR time of propofol anesthesia (Figure 4C). EEG recordings showed that the BSR was increased during propofol anesthesia after the activation of aTRN^SST^ neurons (Figure 4D). Furthermore, similar to aTRN^PV^ neurons, optogenetic activation of aTRN^SST^ neurons caused a decreased δ wave (1–4 Hz) percentage and an increased percentage of α wave (8–12 Hz) and β wave (12–25 Hz) during the RORR stage of propofol anesthesia (Figure 4E–G, Figure S3B,C).

### 3.5 Optogenetic activation of aTRN^SST^ Neurons accelerates the induction and decelerates the emergence of isoflurane anesthesia

**FIGURE 5:**
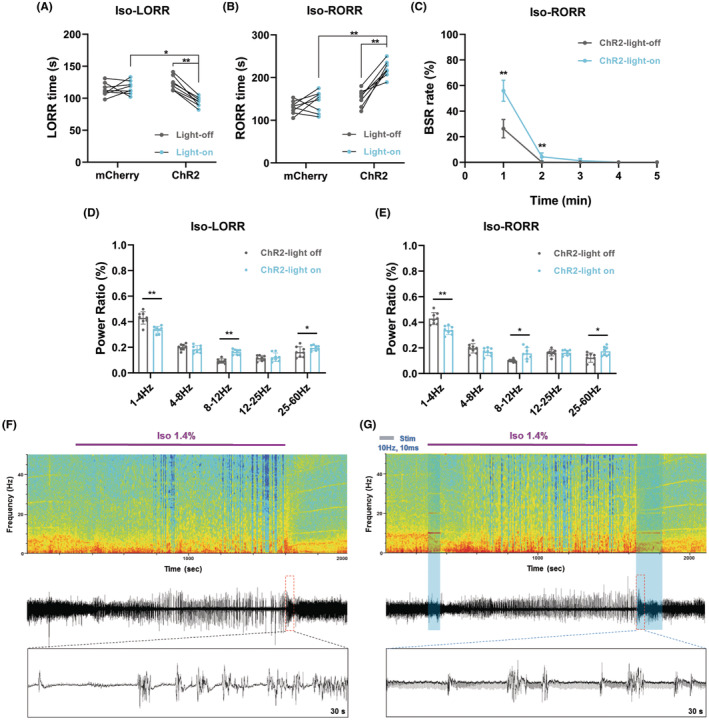
Optogenetic activation of aTRN^SST^ Neurons accelerates the induction and decelerates the emergence of isoflurane anesthesia. (A, B) The effect of aTRN^SST^ neuron activation by blue laser on LORR and RORR time under 1.4% isoflurane anesthesia (LORR: mCherry‐light‐off: 113.37 ± 10.39 s; mCherry‐light‐on: 116.50 ± 10.42 s; ChR2‐light‐off: 122.75 ± 10.91 s; ChR2‐light‐on: 94.25 ± 7.38 s; RORR: mCherry‐light‐off: 130.12 ± 15.18 s; mCherry‐light‐on: 143.13 ± 24.63 s; ChR2‐light‐off: 152.75 ± 19.26 s; ChR2‐light‐on: 219.75 ± 18.46 s; *n* = 8). (C) The effect of aTRN^SST^ neuron activation by blue laser on BSR during the RORR period of 1.4% isoflurane anesthesia (*n* = 8). (D, E) The power distribution of EEG frequency bands in ChR2‐light off or ChR2‐light on group under 1.4% isoflurane anesthesia (*n* = 8). (F, G) Representative EEG wave forms and spectrograms EEG power of ChR2‐light off or ChR2‐light on group under 1.4% isoflurane anesthesia. Paired Student's *t*‐tests and One‐way ANOVA: (A, B). Paired Student's *t*‐tests: (C–E). *p < 0.05, **p < 0.01.

Furthermore, we tested whether activation of aTRN^SST^ neurons affects isoflurane anesthesia (Figure 3A). We found that activation of aTRN^SST^ neurons by blue laser accelerated the LORR time and delayed the RORR time of isoflurane anesthesia (Figure 5A,B). Similar to activation of aTRN^PV^ neurons, activation of aTRN^SST^ neurons also increased the BSR in the emergence period of isoflurane anesthesia (Figure 5C). EEG recordings showed decreased δ wave (1–4 Hz) percentage and increased percentage of α wave (8–12 Hz) and γ wave (25–60 Hz) during both the LORR induction and the emergence period of isoflurane anesthesia in the ChR2‐expressing mice after blue laser stimulation (Figure 5D–G, Figure S3D–G).

Combining the data from optogenetic activation of aTRN^PV^ and aTRN^SST^ neurons, we draw the conclusion that both aTRN^PV^ and aTRN^SST^ neurons promotes the induction of isoflurane anesthesia and delays the recovery of both propofol and isoflurane anesthesia. But the EEG recordings showed that optogenetic activation of aTRN^PV^ and aTRN^SST^ neurons both decreased δ wave (1–4 Hz) proportion, which referred the cortical wakefulness.^44^ This is inconsistent with the promotion effect for anesthesia of these two types of aTRN neurons. To clarify the precise functions of aTRN^PV^ and aTRN^SST^ neurons in anesthesia, chemogenetic tools for continuous manipulation of aTRN neurons were generated for behavior tests and EEG recording.

### 3.6 Chemogenetic activation of aTRN^PV^ Neurons delays the emergence of propofol anesthesia

**FIGURE 6:**
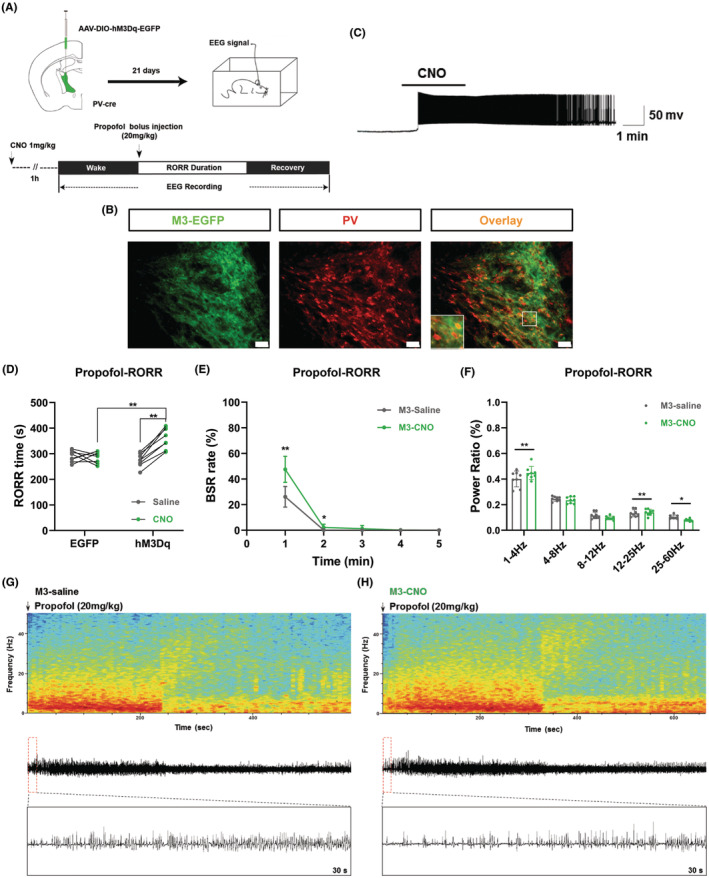
Chemogenetic activation of aTRN^PV^ Neurons delays the emergence of propofol anesthesia. (A) Up: schematic diagram of virus injection into aTRN and EEG electrode implantation. Down: schematic diagram of RORR and EEG recording during propofol anesthesia by single dose of 20 mg/kg injection. (B) Representative images of chemogenetic virus (M3‐EGFP, green) expression in aTRN^PV^ neurons (red, scale bar, 50 μm). (C) Ex vivo electrophysiology of M3 virus action in aTRN^PV^ neurons. (D) The effect of aTRN^PV^ neuron activation by CNO on RORR time under propofol anesthesia (EGFP‐saline: 290.13 ± 22.20 s; EGFP‐CNO: 286.38 ± 22.60 s; M3‐saline: 275.00 ± 26.07 s; M3‐CNO: 360.38 ± 40.53 s; *n* = 8). (E) The effect of aTRN^PV^ neuron activation by CNO on BSR during the RORR period of propofol anesthesia (*n* = 8). (F) The power distribution of EEG frequency bands in M3‐saline or M3‐CNO group under propofol anesthesia (*n* = 8). (G, H) Representative EEG wave forms and spectrograms EEG power of M3‐saline or M3‐CNO group under propofol anesthesia. Paired Student's *t*‐tests and One‐way ANOVA: (D). Paired Student's *t*‐tests: (E, F). *p < 0.05, **p < 0.01.

Previous studies and our data revealed that aTRN is mainly composed of PV^+^ GABAergic neurons,^45^ and we found shared roles of aTRN^PV^ and aTRN^SST^ neurons in anesthesia. So we used PV‐IRES‐cre mice to study the effect of chemogenetic activation of aTRN neurons on propofol and isoflurane anesthesia. To continuously modulate the activity of aTRN^PV^ neurons, rAAV‐EF1α‐DIO‐hM3Dq‐EGFP (M3) virus was unilaterally microinjected into aTRN of the PV‐IRES‐cre mice (Figure 6A). Immunofluorescence staining confirmed virus expression in the aTRN^PV^ neurons (Figure 6B and Figure S4A). One hour before propofol application, CNO was intraperitoneally injected to activate the aTRN^PV^ neurons. The electrophysiology recording on the brain slice showed increased firing of aTRN^PV^ neurons after CNO injection in the M3‐expressing mice (Figure 6C). Activation of aTRN^PV^ neurons by CNO significantly prolonged the emergence time (RORR) after 20 mg/kg propofol injection (Figure 6D). In the M3‐expressing mice, activation of aTRN^PV^ neurons by CNO also increased the BSR during the RORR period of propofol anesthesia (Figure 6E). On the contrary of optogenetic activation of aTRN^PV^ neurons, TRN^PV^ neuron activation by CNO increased the percentage of the δ wave (1–4 Hz) and decreased the γ wave (25–60 Hz) proportion during the RORR period of propofol anesthesia (Figure 6F–H, Figure S4B,C), which is consistent with the delay effect for emergence of propofol anesthesia.

### 3.7 Chemogenetic activation of aTRN^PV^ neurons promotes the induction and delays the emergence of isoflurane anesthesia

**FIGURE 7:**
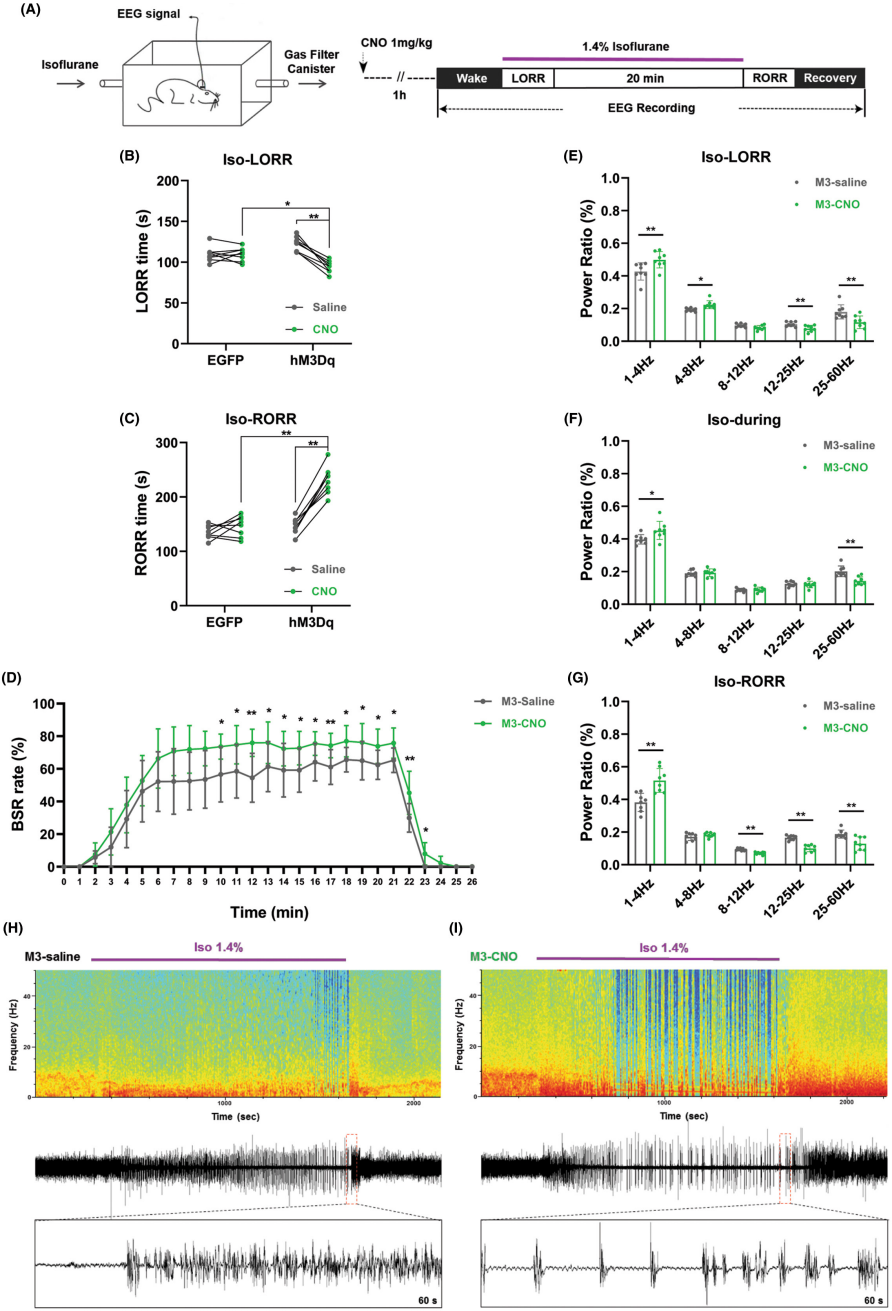
Chemogenetic activation of aTRN^PV^ neurons promotes the induction and delays the emergence of isoflurane anesthesia. (A) Schematic diagram of LORR, RORR and EEG recording during 1.4% isoflurane anesthesia. (B, C) The effect of aTRN^PV^ neuron activation by CNO on LORR and RORR time under 1.4% isoflurane anesthesia (LORR:EGFP‐saline: 109.63 ± 9.35 s; EGFP‐CNO: 109.63 ± 8.30s; M3‐saline: 122.37 ± 8.98 s; M3‐CNO: 94.25 ± 7.38 s; RORR:EGFP‐saline: 131.13 ± 12.39 s; EGFP‐CNO: 146.37 ± 19.05 s; M3‐saline: 146.37 ± 15.11 s; M3‐CNO: 230.75 ± 25.79 s; *n* = 8). (D) The effect of aTRN^PV^ neuron activation by CNO on BSR during 1.4% isoflurane anesthesia (*n* = 8). (E–G) The power distribution of EEG frequency bands in M3‐saline or M3‐CNO group under 1.4% isoflurane anesthesia (*n* = 8). (H, I) Representative EEG wave forms and spectrograms EEG power of M3‐saline or M3‐CNO group under 1.4% isoflurane anesthesia. Paired Student's *t*‐tests and One‐way ANOVA: (B, C). Paired Student's *t*‐tests: (D–G). *p < 0.05, **p < 0.01.

During isoflurane anesthesia, shortened LORR time and prolonged RORR time were observed in the CNO treated M3‐expressing mice compared to the M3‐saline and EGFP‐CNO groups (Figure 7A–C). In the M3‐expressing mice, activation of aTRN^PV^ neurons by CNO administration significantly increased the BSR during the maintenance and emergence period of isoflurane anesthesia (Figure 7D). Furthermore, EEG recordings showed a higher δ wave (1–4 Hz) proportion and a lower percentage of γ wave (25–60 Hz) during the whole progression of isoflurane anesthesia in CNO treated M3‐expressing mice, including induction, maintenance and emergence period (Figure 7E–I, Figure S4D–I). These results indicate that continuous activation of aTRN^PV^ neurons accelerates isoflurane induction and delays the recovery of both propofol and isoflurane anesthesia.

Combining the data from optogenetic and chemogenetic activation of aTRN neurons, we conclude that activation of aTRN GABAergic neurons promotes the induction of isoflurane anesthesia and delays the recovery of propofol/isoflurane anesthesia.

### 3.8 Manipulation of aTRN^VGAT^ neurons regulates the behavior of propofol and isoflurane anesthesia

**FIGURE 8:**
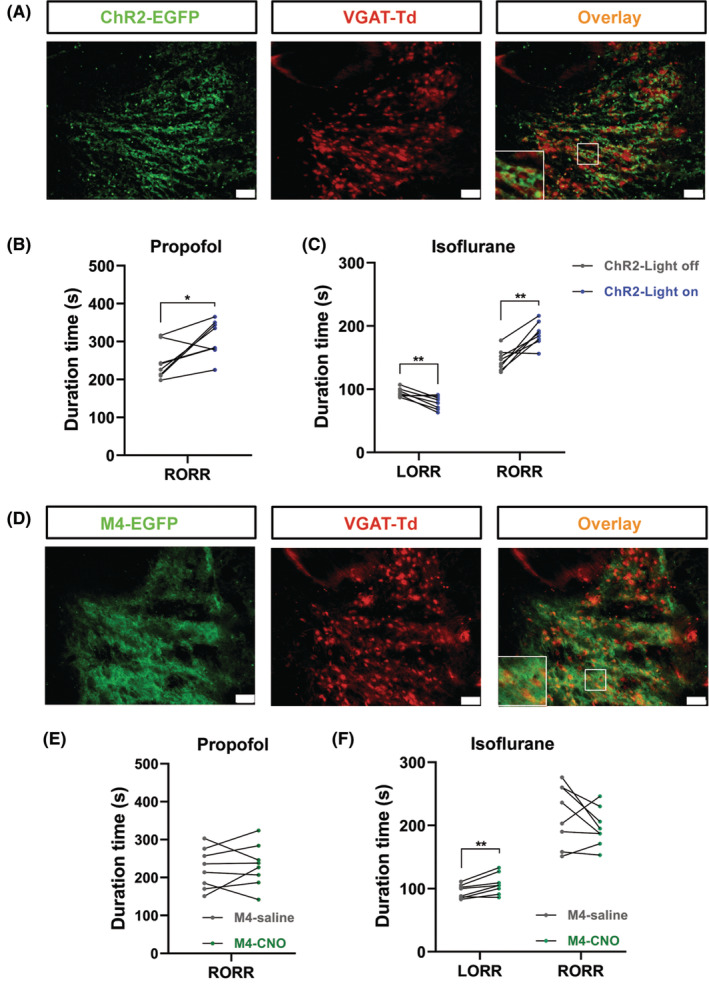
Manipulation of aTRN^VGAT^ neurons regulates the behavior of propofol and isoflurane anesthesia. (A) Representative images of optogenetic virus (ChR2‐EGFP, green) expression in TRN^VGAT^ neurons (red, scale bar, 50 μm). (B) The effect of aTRN^VGAT^ neuron activation by blue laser on RORR times under propofol anesthesia (*n* = 8). (C) The effect of aTRN^VGAT^ neuron activation by blue laser on LORR and RORR times under 1.4% isoflurane anesthesia (*n* = 8). (D) Representative images of chemogenetic virus (M4‐EGFP, green) expression in aTRN^VGAT^ neurons (red, scale bar, 50 μm). (E) The effect of aTRN^VGAT^ neuron inhibition by CNO on RORR times under propofol anesthesia (*n* = 8). (F) The effect of aTRN^VGAT^ neuron inhibition by CNO on LORR and RORR times under 1.4% isoflurane anesthesia (*n* = 8). Paired Student's *t*‐tests: (B, C, E, F). *p < 0.05, **p < 0.01.

Then we used VGAT‐IRES‐cre mice to study the effect of the whole mount aTRN GABAergic neurons manipulation on propofol and isoflurane anesthesia. rAAV‐EF1α‐DIO‐hChR2‐EGFP virus (ChR2) was unilaterally injected into aTRN of the VGAT‐IRES‐cre mice, followed placement of an optical fiber in the same position (Figure 8A). Optogenetic activation of aTRN^VGAT^ neurons prolonged the RORR time of propofol anesthesia (Figure 8B). Accelerated the LORR time and delayed the RORR time were also observed after activation of aTRN^VGAT^ neurons during isoflurane anesthesia (Figure 8C). Next, we performed the chemogenetic inhibition strategy to confirm the role of aTRN^VGAT^ neurons in GA. rAAV‐EF1α‐DIO‐hM4Di‐EGFP (M4) virus was bilaterally microinjected into aTRN of the VGAT‐IRES‐cre mice (Figure 8D). One hour before propofol application, CNO was intraperitoneally injected to deactivate the aTRN^VGAT^ neurons. Interestingly, we only found the prolonged LORR time of isoflurane anesthesia (Figure 8F), the RORR time of propofol or isoflurane anesthesia was not significantly altered (Figure 8E,F).

At last, to test which part of TRN regulates anesthesia progression, we injected IBO into aTRN or the posterior sector of the TRN (pTRN) of the VGAT‐td mice to kill aTRN or pTRN neurons, respectively. Seven days after IBO injection, the number of tdTomato‐labeled aTRN or pTRN neurons was massively decreased in the lesion group (Figure S5A,B,E,F). We found a shorter RORR time in the aTRN lesion mice in propofol anesthesia (Figure S5C). Longer LORR time and shorter RORR time were observed in the aTRN lesion mice during isoflurane anesthesia (Figure S5D). On the contrary, pTRN lesion did not alter the behaviors of propofol or isoflurane anesthesia (Figures S5G,H). These data suggest that aTRN may be the essential region of TRN for the regulation of GA.

## 4 DISCUSSION

In this study, we set up immunofluorescence, optogenetic, chemogenetic and EEG recording techniques to investigate the roles of the aTRN GABAergic neurons in regulating the process of propofol/isoflurane anesthesia. PV/SST double staining showed that aTRN is composed of PV and SST subtypes of GABAergic neurons. c‐Fos imaging showed that the activity of both aTRN^PV^ and aTRN^SST^ neurons was increased during propofol/isoflurane induced anesthesia and decreased during recovery period after anesthesia. Optogenetic activation of both aTRN^PV^ and aTRN^SST^ neurons promoted the induction of isoflurane anesthesia and delayed the emergence of both propofol and isoflurane anesthesia. Accordingly, Optogenetic activation of aTRN^PV^ and aTRN^SST^ neurons accumulated the BSR during propofol/isoflurane induced anesthesia. Additionally, chemogenetic activation of aTRN^PV^ neurons also accelerated the induction time of isoflurane anesthesia and prolonged the emergence time of propofol/isoflurane anesthesia and increased the BSR during propofol/isoflurane induced anesthesia. Furthermore, the power distribution of EEG frequency is different under optogenetic and chemogenetic activation of aTRN neurons, respectively, which may attribute to the specific role of aTRN neurons on cortical spindles. Taken together, these findings suggest a critical role of aTRN in controlling the process of propofol‐ and isoflurane‐mediated anesthesia.

The TRN is the only GABAergic neurons formed neural nucleus in the thalamus, which is the main inhibition source for the thalamus.^23^, ^24^ The TRN was reported to include almost PV‐expressing GABAergic neurons, which was considered as a homogeneous GABAergic neuron formed nucleus.^46^, ^47^ But SST‐expressing neurons were identified in TRN recently,^45^, ^48^ and TRN neurons have been reported to exhibit heterogeneities in molecular and electrophysiological properties, and neural connectivity.^28^, ^43^, ^45^, ^48^, ^49^ These finding indicated possible distinct functions of different subtypes of TRN GABAergic neurons. However, inconsistent results have been reported in the previous studies. Clemente‐Perez et al.^48^ reported that PV‐ and SST‐expressing cells are two distinct distinguished subtypes of the TRN neurons, whereas Li et al.^43^ showed that nearly all TRN neurons expressed PV and about 64% of them expressed SST, which suggested the overlapping of STT and partly PV‐expressing neurons in the TRN. To confirm the composition of TRN GABAergic neurons, we proceeded PV/SST double staining and found that about 40% of aTRN neurons are expressing both PV and SST, whereas the proportion of PV and SST neurons in aTRN is around 80% and 60% respectively (Figure S1), which is consistent with the Li's article.^43^ Furthermore, c‐Fos staining revealed that both aTRN^PV^ and aTRN^SST^ neurons were activated during propofol/isoflurane induced anesthesia (Figure 1). These data indicated the involvement of both aTRN^PV^ and aTRN^SST^ neurons in regulation of GA.

We next generated optogenetic and chemogenetic tools to figure out the function of aTRN^PV^ and aTRN^SST^ neurons in GA. As predicted, optogenetic activation of both aTRN^PV^ and aTRN^SST^ neurons or chemogenetic activation of aTRN^PV^ neurons promoted the induction of isoflurane anesthesia and delayed the emergence of both propofol and isoflurane anesthesia accompanied with the accumulated BSR during anesthesia. This is consistent with the former researches which showed that TRN activation induces non‐rapid‐eye‐movement sleep (NREM) and decreases arousal.^50^, ^51^ Interestingly, optogenetic or chemogenetic activation of aTRN neurons showed different effects on the power distribution of EEG frequency during anesthesia. Optogenetic activation of aTRN^PV^ and aTRN^SST^ neurons caused decreased δ wave (1–4 Hz) proportion and increased percentage of α wave (8–12 Hz) and γ wave (25–60 Hz) during propofol/isoflurane anesthesia, whereas δ wave (1–4 Hz) is the characteristic of EEG of GA. On the contrary, chemogenetic activation of aTRN^PV^ neurons increased the percentage of the δ wave (1–4 Hz) and decreased the γ wave (25–60 Hz) proportion during anesthesia, which is consistent with the promotion effect of aTRN^PV^ neurons on the induction of GA. The opposite effects of optogenetic and chemogenetic activation of aTRN neurons on EEG structures during GA may due to the specific regulation of TRN for spindles (7–16 Hz) and α oscillations (8–12 Hz) in the cortex.^25^, ^28^, ^32^, ^51^, ^52^, ^53^, ^54^, ^55^, ^56^ TRN neurons are identified to generate spindle oscillations during sleep^32^, ^50^, ^51^, ^54^, ^56^ and sustained stimulation of TRN also increases δ wave (1–4 Hz) related slow‐wave sleep.^51^ Consequently, we observed the increased δ wave (1–4 Hz) proportion during propofol/isoflurane anesthesia after aTRN^PV^ neuron activation by chemogenetic approaches, which may contribute to the promotion for anesthesia. In the other hand, as the pacemaker of the cortical spindles, the TRN is served as a rhythmic nucleus in the thalamus,^50^, ^54^ so blue‐light stimulation with distinct frequency on aTRN^PV^ or aTRN^SST^ neurons may generate the cortical EEG band in specific frequency. In our optogenetic experiments, 10 Hz blue‐light stimulation on both aTRN^PV^ and aTRN^SST^ neurons caused massive accumulation of 10 Hz EEG band (Figures 2H, 3H, 4G and 5G), which resulted in the increased percentage of α wave (8–12 Hz). The decreased δ wave (1–4 Hz) proportion may be the compensation in response to α wave increase. Besides the different effects on EEG composition, optogenetic and chemogenetic activation of aTRN neurons suggested the shared roles of aTRN^PV^ and aTRN^SST^ neurons in regulation of propofol/isoflurane anesthesia.

In our study, we did not see the different functions of aTRN^PV^ and aTRN^SST^ neurons in GA, although the PV and SST‐expressing neurons in the TRN were reported to play different roles in many other TRN‐related procedures.^48^ The possible reason is the overlapping of the PV and SST‐expression in the aTRN neurons. Our data showed that about 40% of the aTRN neurons express both PV and SST. So the manipulation of aTRN neurons using PV‐IRES‐cre and SST‐ IRES‐cre mice is hardly for us to distinguish the distinct roles of aTRN^PV^ and aTRN^SST^ neurons in GA. In the other hand, the PV and SST‐expressing TRN neurons exhibited different electrophysiological properties and formed distinct neural circuits,^43^, ^45^, ^48^ which indicated the distinguished mechanism of aTRN^PV^ and aTRN^SST^ neurons in the regulation of GA, even though they showed similar promotion effect on anesthesia induction. At last Li et al.^43^ suggested that the molecular and electrophysiological properties may be a more appropriate standard for classification of the TRN neurons rather than the simply identification of PV or SST expression. In the future, an activity dependent tool for neuron manipulation, such as the TRAP (targeted recombination in active populations),^57^ may be useful to clarify the exact functions of aTRN^PV^ and aTRN^SST^ neurons in GA.

To further confirm the roles of aTRN GABAergic neurons in GA, we used VGAT‐IRES‐cre to manipulate the whole mount of the aTRN neurons. ChR2‐induced activation of aTRN^VGAT^ neurons showed comparable effects on propofol and isoflurane anesthesia to aTRN^PV^ and aTRN^SST^ neurons (Figure 8A–C), which suggested the shared mechanism of different subtypes of aTRN GABAergic neurons in GA. Interestingly, M4‐mediated inhibition of aTRN^VGAT^ neurons only showed influence on the induction of isoflurane anesthesia (Figure 8F), but not the recovery of propofol or isoflurane anesthesia (Figure 8E,F). This may be attributed to the accumulated activity of TRN neurons during anesthesia and the activity recovery during the emergency period. In the ChR2 experiment, continuous activation of aTRN^VGAT^ neurons during the RORR stage sustained the maintenance of anesthesia to delay the recovery. On the contrary, M4‐mediated inhibition of aTRN^VGAT^ neurons blunted the activation of aTRN neurons, which is critical for the induction and maintenance of anesthesia.

Taken together, our data suggest that aTRN GABAergic neurons may involve in the regulation of distinct stages of GA.

## AUTHOR CONTRIBUTIONS

All authors contributed to the experimental conception and design of the study. Material preparation, data collection, and analyses were performed by R.Y., S.C., F.Z., Y.Y., L.Z. and Y.Z. R.Y., S.C. and D.L. generated all the mouse models. The manuscript was written by L.Z., Y.Z., H.W. and T.Y., and all authors commented on the previous versions of the manuscript. All authors have read and approved the final manuscript.

## CONFLICT OF INTEREST STATEMENT

The authors declare that they have no conflicts of interest in this work.

## Supporting information


Figures S1–S5.

## Data Availability

All data reported in this paper will be shared by the corresponding author upon request.
