# Supplementary material for: GABAergic neurons of anterior thalamic reticular nucleus regulate states of consciousness in propofol‐ and isoflurane‐mediated general anesthesia
Source: CNS Neurosci Ther. 2024 Jun 3;30(6):e14782. doi: 10.1111/cns.14782 (PMC11145368; doi:10.1111/cns.14782)
Supplement: Supplementary file 1 — Figures S1–S5. [file CNS-30-e14782-s001.docx]

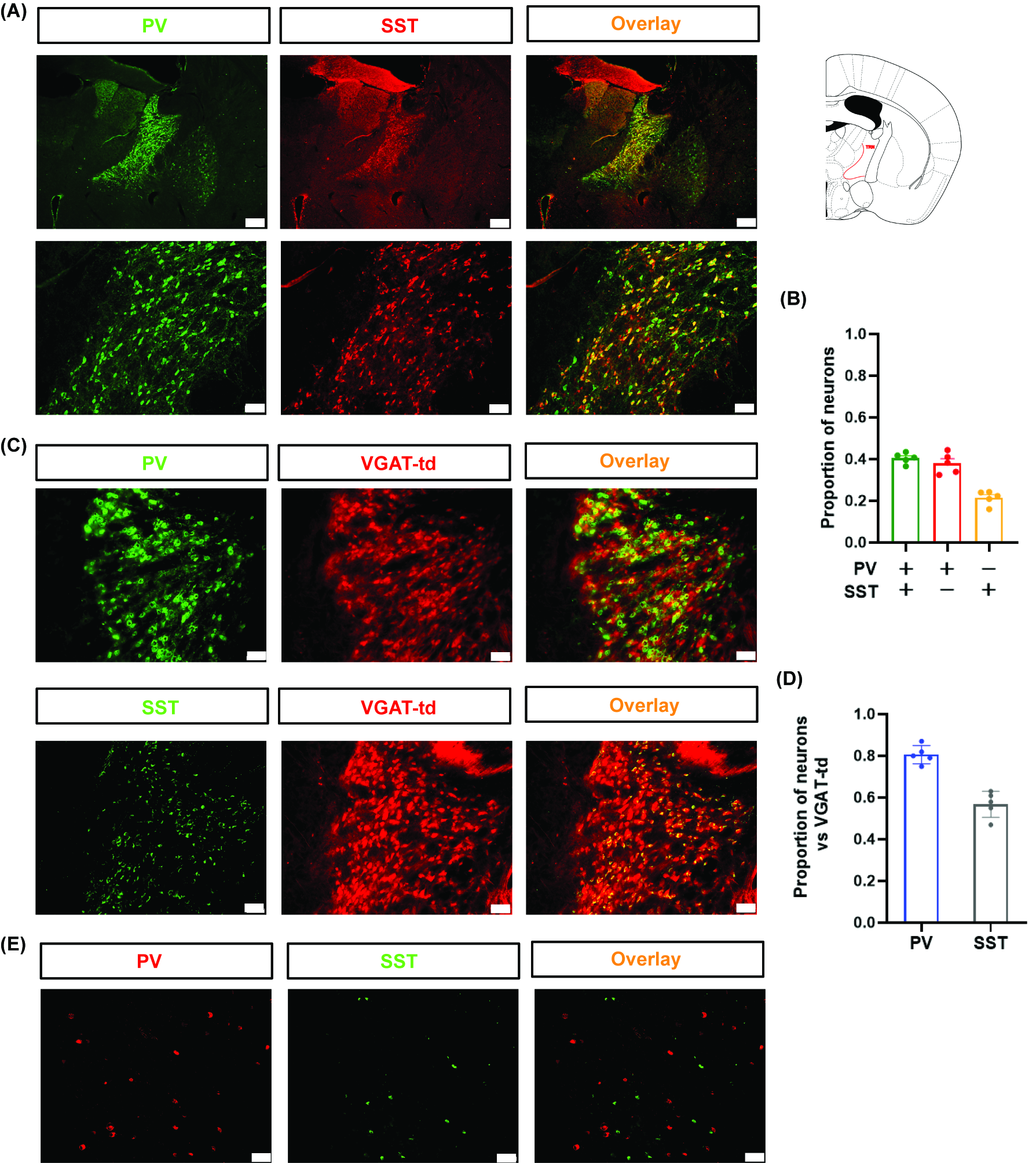


**FIGURE S1. PV and SST expression in the GABAergic neurons of aTRN.** (A) Representative images of PV/SST double-staining in aTRN of C57/B6 mice (up: scale bar, 200 μm; down: scale bar, 50 μm). (B) The proportion of PV and SST neurons in aTRN, respectively (n=5). (C) Representative images of PV/SST expression in aTRN of VGAT-td mice (scale bar, 50 μm). (D) The proportion of PV and SST neurons in VGAT-td labeled neurons of aTRN, respectively (n=5). (E) Representative images of PV/SST double-staining in mPFC of C57/B6 mice (scale bar, 50 μm).


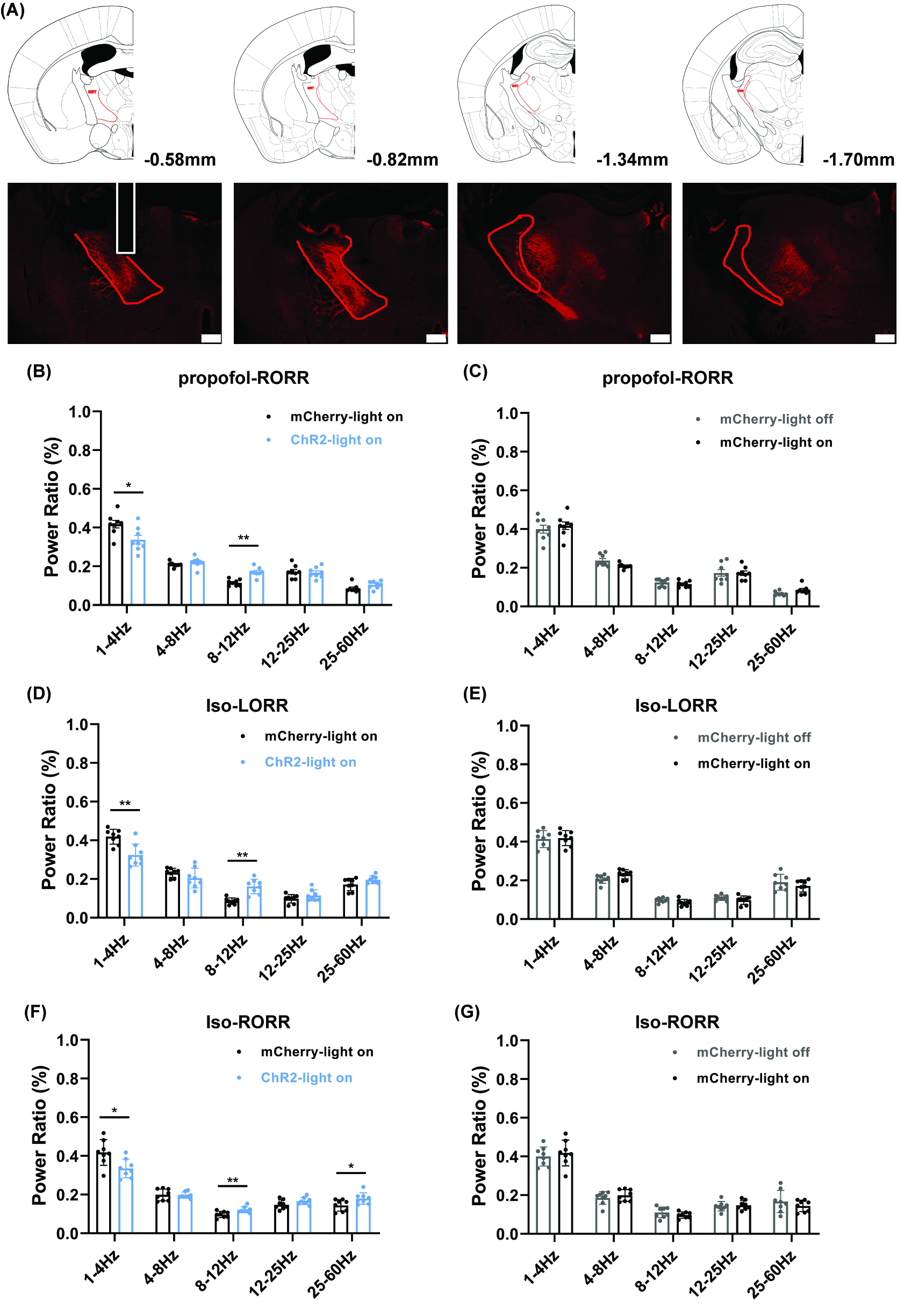


**FIGURE S2. EEG recording of Ctrl or ChR2-expressing aTRN^PV^ mice during propofol and isoflurane anesthesia.** (A) Representative images of ChR2-mCherry virus expression in TRN of PV-IRES-cre mice (scale bar, 50 μm). (B) The power distribution of EEG frequency bands compared with mCherry-light-on and ChR2-light-on group during the emergence after propofol anesthesia (n=8). (C) The power distribution of EEG frequency bands compared with mCherry -light-off and mCherry-light-on group during the emergence after propofol anesthesia (n=8). (D) The power distribution of EEG frequency bands compared with mCherry-light-on and ChR2-light-on group during 1.4% isoflurane anesthesia induction (n=8). (E) The power distribution of EEG frequency bands compared with mCherry -light-off and mCherry-light-on group during 1.4% isoflurane anesthesia induction (n=8). (F) The power distribution of EEG frequency bands compared with mCherry-light-on and ChR2-light-on group during the emergence after 1.4% isoflurane anesthesia (n=8). (G) The power distribution of EEG frequency bands compared with mCherry -light-off and mCherry-light-on group during the emergence after 1.4% isoflurane anesthesia (n=8). One-way ANOVA: (B, D, F). Paired Student’s t-tests: (C, E, G). **p*< 0.05, ***p*< 0.01.


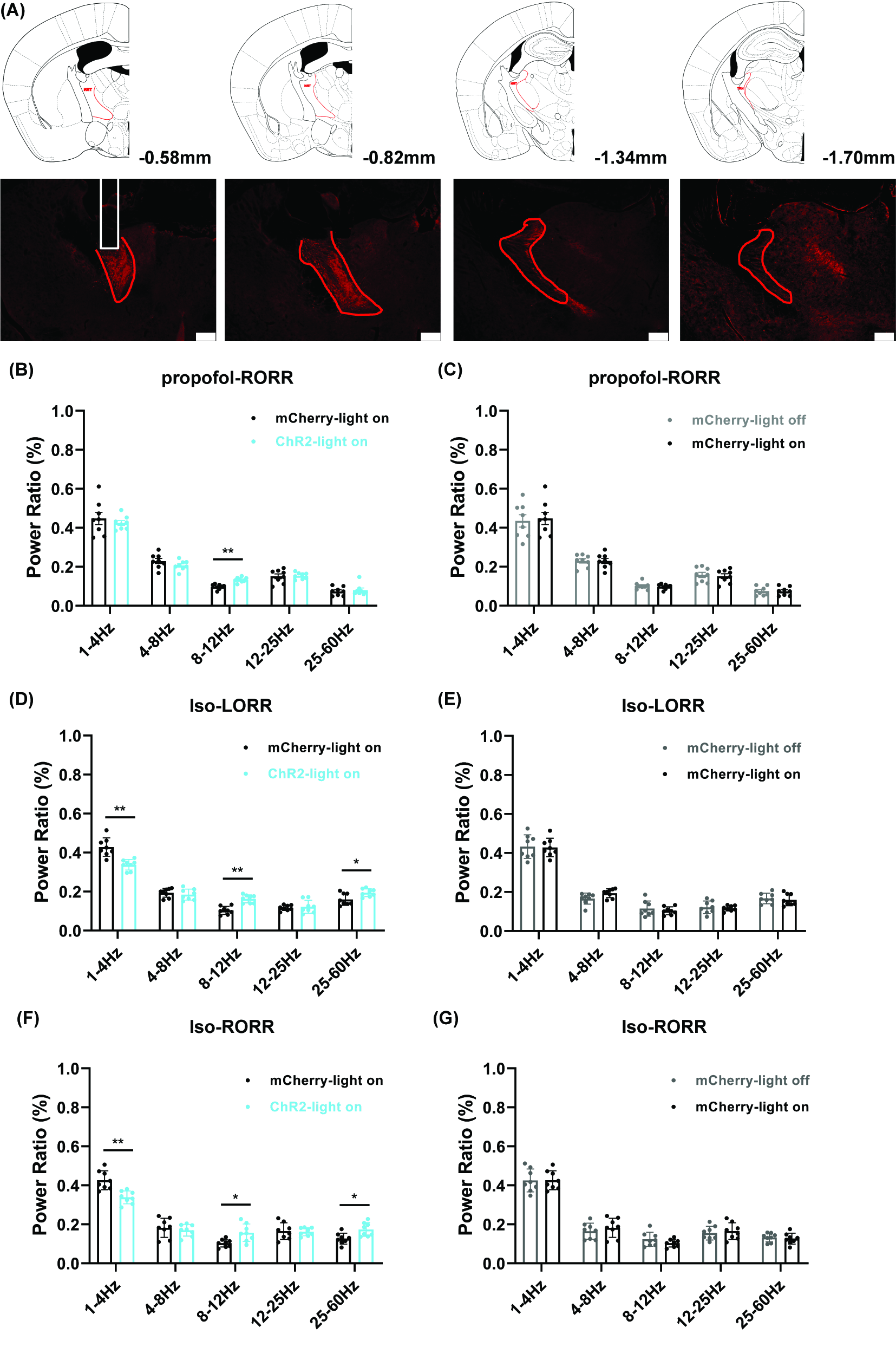


**FIGURE S3. EEG recording of Ctrl or ChR2-expressing aTRN^SST^ mice during propofol and isoflurane anesthesia.** (A) Representative images of ChR2-mCherry virus expression in TRN of SST-IRES-cre mice (scale bar, 50 μm). (B) The power distribution of EEG frequency bands compared with mCherry-light-on and ChR2-light-on group during the emergence after propofol anesthesia (n=8). (C) The power distribution of EEG frequency bands compared with mCherry -light-off and mCherry-light-on group during the emergence after propofol anesthesia (n=8). (D) The power distribution of EEG frequency bands compared with mCherry-light-on and ChR2-light-on group during 1.4% isoflurane anesthesia induction (n=8). (E) The power distribution of EEG frequency bands compared with mCherry -light-off and mCherry-light-on group during 1.4% isoflurane anesthesia induction (n=8). (F) The power distribution of EEG frequency bands compared with mCherry-light-on and ChR2-light-on group during the emergence after 1.4% isoflurane anesthesia (n=8). (G) The power distribution of EEG frequency bands compared with mCherry -light-off and mCherry-light-on group during the emergence after 1.4% isoflurane anesthesia (n=8). One-way ANOVA: (B, D, F). Paired Student’s t-tests: (C, E, G). **p*< 0.05, ***p*< 0.01.


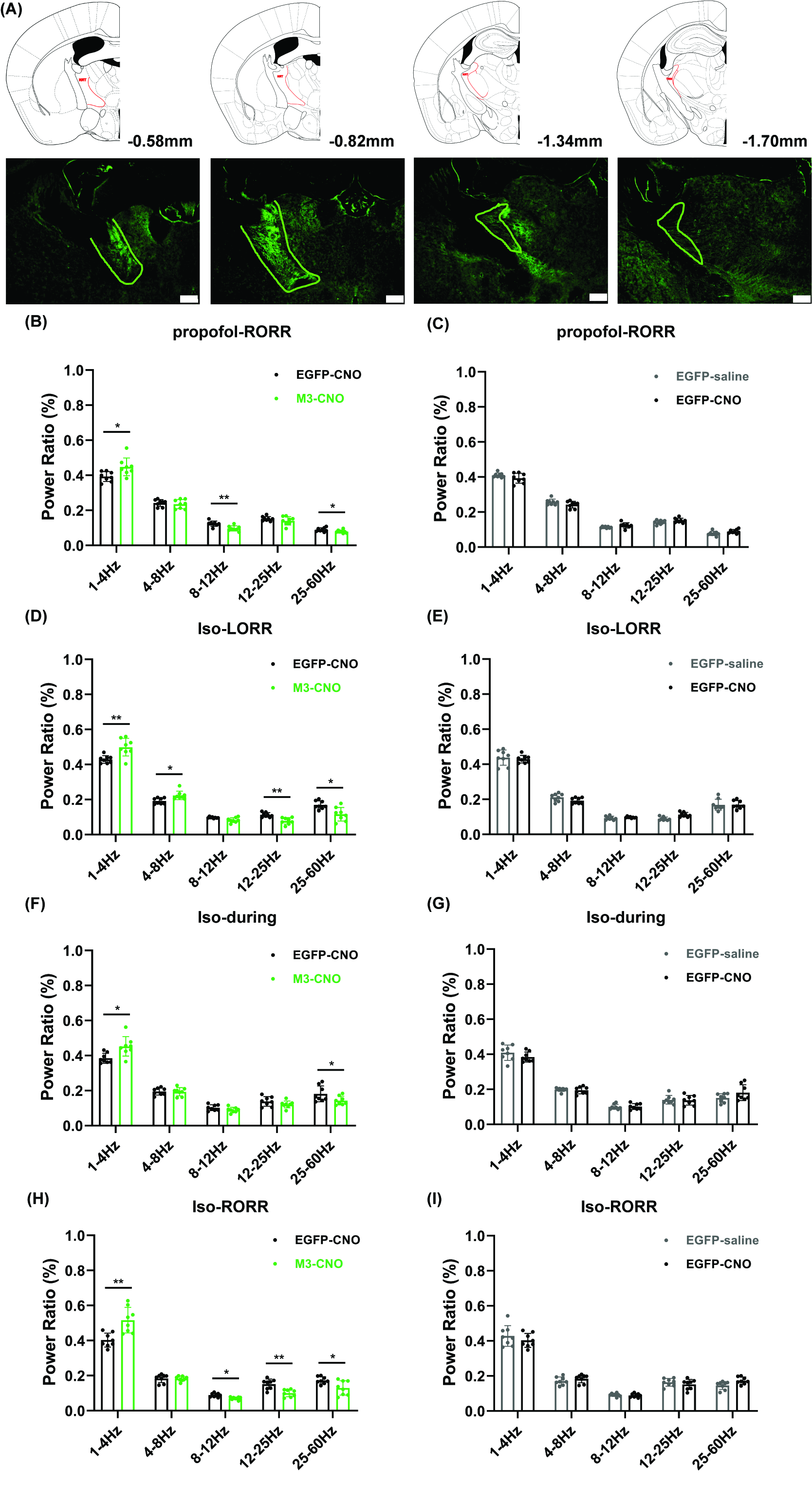


**FIGURE S4. EEG recording of Ctrl or M3-expressing aTRN^PV^ mice during propofol and isoflurane anesthesia.** (A) Representative images of M3-EGFP virus expression in TRN of PV-IRES-cre mice (scale bar, 50 μm). (B) The power distribution of EEG frequency bands compared with mCherry-light-on and ChR2-light-on group during the emergence after propofol anesthesia (n=8). (C) The power distribution of EEG frequency bands compared with mCherry -light-off and mCherry-light-on group during the emergence after propofol anesthesia (n=8). (D) The power distribution of EEG frequency bands compared with mCherry-light-on and ChR2-light-on group during 1.4% isoflurane anesthesia induction (n=8). (E) The power distribution of EEG frequency bands compared with mCherry -light-off and mCherry-light-on group during 1.4% isoflurane anesthesia induction (n=8). (F) The power distribution of EEG frequency bands compared with mCherry-light-on and ChR2-light-on group during 1.4% isoflurane anesthesia maintenance (n=8). (G) The power distribution of EEG frequency bands compared with mCherry -light-off and mCherry-light-on group during 1.4% isoflurane anesthesia maintenance (n=8). (H) The power distribution of EEG frequency bands compared with mCherry-light-on and ChR2-light-on group during the emergence after 1.4% isoflurane anesthesia (n=8). (I) The power distribution of EEG frequency bands compared with mCherry -light-off and mCherry-light-on group during the emergence after 1.4% isoflurane anesthesia (n=8). One-way ANOVA: (B, D, F, H). Paired Student’s t-tests: (C, E, G, I). **p*< 0.05, ***p*< 0.01.

**
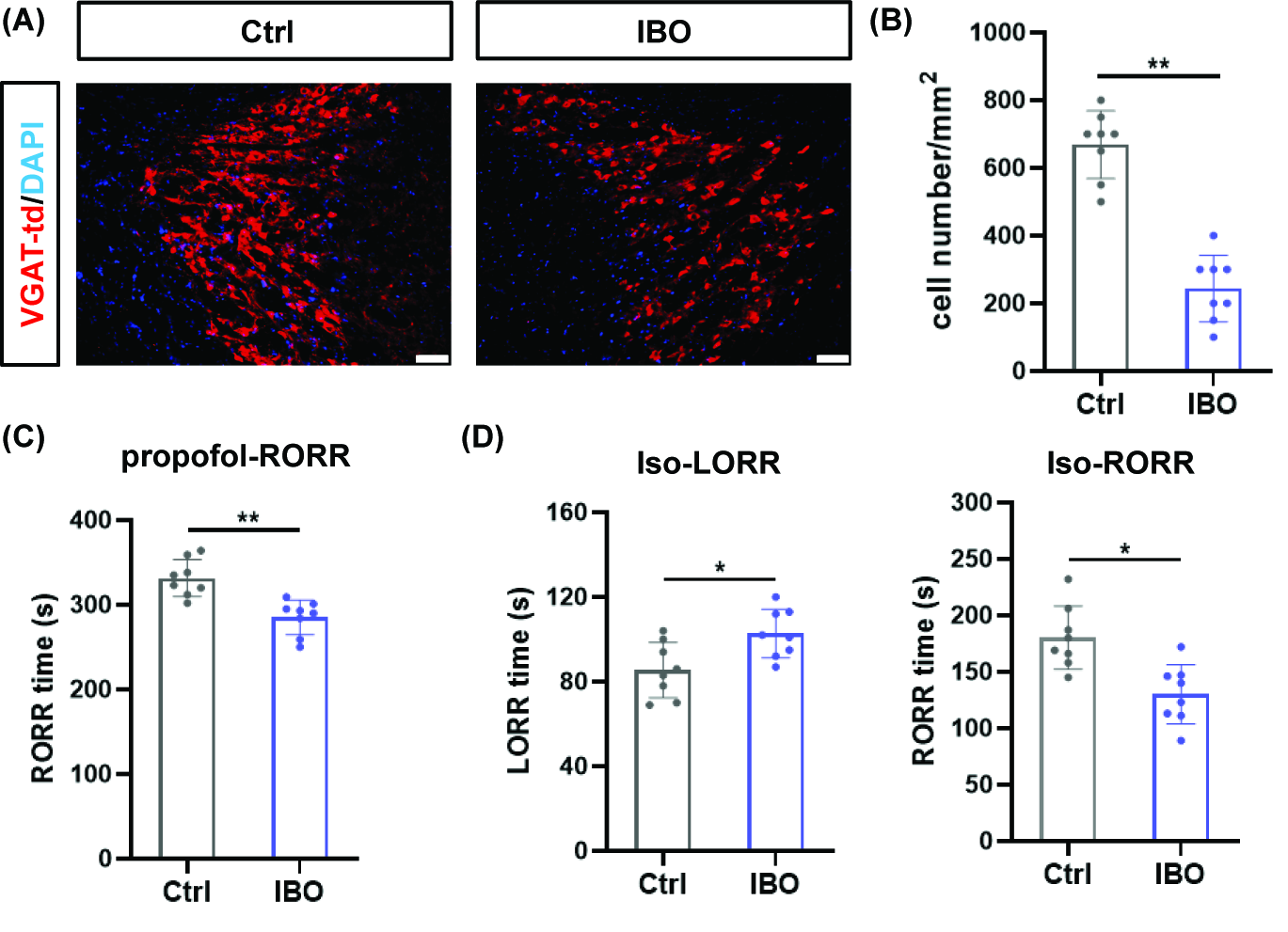
**

**FIGURE S5. Effects of chemical lesion of aTRN neurons on propofol and isoflurane anesthesia.** (A) Representative images of VGAT-td labeled aTRN neurons in Ctrl and lesion groups in VGAT-td mice (scale bar, 50 μm). (B) Quantification of VGAT-td cell number (per mm^2^) in aTRN (Ctrl: 669±100; IBO: 248±98; n=8). (C) The effect of aTRN lesion on RORR time under propofol anesthesia (Ctrl: 331.63±21.78s; IBO: 285.13±20.43s; single dose injection of 20 mg/kg, n=8). (D) The effect of aTRN lesion on LORR and RORR time under 1.4% isoflurane anesthesia (LORR: Ctrl: 85.50±13.09s; IBO: 102.75±11.44s; RORR: Ctrl: 172.88±35.15s; IBO: 130.13±26.16s; n=8). Unpaired Student’s t-tests: (B-D). **p*< 0.05, ***p*< 0.01.
